# Editorial for the Special Issue on Advancements in Plant Photobiology

**DOI:** 10.3390/plants14243731

**Published:** 2025-12-07

**Authors:** Robert Luciński

**Affiliations:** Department of Plant Physiology, Institute of Experimental Biology, Faculty of Biology, Adam Mickiewicz University in Poznań, ul Uniwersytetu Poznańskiego 6, 61-614 Poznań, Poland; rtl@amu.edu.pl

Light constitutes a fundamental environmental factor that profoundly influences plant growth, development, and metabolism. Red light plays a pivotal role in light-dependent photosynthetic reactions and regulates key developmental processes, including stem elongation, leaf expansion, and flowering. In contrast, blue light is primarily involved in the regulation of phototropism, chloroplast relocation, and stomatal conductance [1].

Plant photobiology represents a scientific discipline dedicated to elucidating the mechanisms by which plants perceive and respond to light. This field encompasses investigations into a wide range of phenomena, including photoperiodism, the effects of light quality and quantity on plant growth and morphogenesis, and the mechanisms underlying photosynthesis. Furthermore, plant photobiology addresses the molecular and physiological bases of light signal transduction pathways and the adaptive responses of plants to diverse light environments.

The articles presented in this Special Issue collectively explore nearly all of these dimensions of plant photobiology, contributing novel and significant insights that deepen our understanding of plant growth, developmental regulation, and the mechanisms facilitating acclimation to fluctuating light conditions. These findings provide a robust theoretical foundation for the development of practical applications aimed at enhancing agricultural productivity while ensuring environmental sustainability.

## 1. Photosynthetic Activity and Adaptations of the Photosynthetic Apparatus to Changing Environmental Conditions

The photosynthetic activity of plants is directly dependent on both the intensity and the quality of light reaching the leaf surface. Plants are constantly exposed to fluctuations in light intensity, which significantly affect their growth and productivity. Potassium ions appear to play an important role in the acclimation of the photosynthetic apparatus to dynamically changing light environments [2]. Low potassium availability adversely affects the photosynthetic performance of *Phaseolus vulgaris* L. under fluctuating light conditions. Under such circumstances, the stimulation of enzymes such as Rubisco may represent an effective strategy to enhance dynamic photosynthetic performance [2].

## 2. Rubisco Activity and CO_2_ Assimilation

Rubisco activity and CO_2_ assimilation are conditioned, among other factors, by the availability of ATP and NADPH, whose efficient production depends on the proper functioning of the photosynthetic electron transport chain [3], as well as on the presence and synthesis of photosynthetic pigments, such as chlorophylls, and their incorporation into the protein complexes forming the main light-harvesting antennae of photosystems, namely LHCII and LHCI. In this context, the chloroplast signal recognition particle components, cpSRP43 and cpSRP54, play a crucial role in directing light-harvesting chlorophyll-binding proteins to the thylakoid membranes.

A comprehensive understanding of the function of cpSRP components is therefore essential for elucidating the mechanisms regulating photosynthesis. The study by [4] demonstrated that both cpSRP43 and cpSRP54 play key roles in chloroplast pigment biosynthesis and significantly contribute to overall chloroplast functionality, although the accumulation of these proteins appears to be subject to distinct regulatory control.

## 3. Regulation of CO_2_ Assimilation Rate

The rate of CO_2_ assimilation is, as expected, closely dependent on the intensity of light reaching the leaf surface. However, the mechanisms underlying this relationship remain relatively poorly understood. Recent studies presented in this Special Issue indicate that the rate of CO_2_ assimilation is largely regulated by the carboxylation capacity of Rubisco (Vₘₐₓ), which in turn depends on energy availability [3]. Characterizing these relationships provides valuable insights into the potential for improving crop performance through the manipulation of Vₘₐₓ.

## 4. Photoprotective Mechanisms and the Role of Chloroplast Proteases

The photosynthetic apparatus, particularly the pigment–protein complexes embedded in the thylakoid membranes that are responsible for harvesting light energy and transferring it to the reaction centers of the photosystems, is especially vulnerable to damage caused by various environmental stresses, most notably by excessive light intensity. In this context, mechanisms protecting the photosynthetic machinery against photoinhibition and photodestruction play a crucial role.

An important component of this complex network of protective and repair systems is chloroplast proteases. Some of them, referred to as intramembrane proteases, e.g., the S2P2 protease, are thought to be involved in the response of the photosynthetic apparatus already at the level of chloroplast gene expression, regulating the synthesis of key photosynthetic proteins. As demonstrated by [5], the S2P2 protease plays a significant role in shaping the stoichiometric relationships among photosystem II core proteins and influences the chloroplast’s capacity to mitigate excessive levels of reactive oxygen species generated under high light stress conditions.

## 5. High-Temperature Stress and Its Impact on Photosynthetic Efficiency

Another major environmental factor, alongside excessive light intensity, that negatively affects photosynthetic efficiency is high temperature. This stress factor has become increasingly pronounced in recent years, coinciding with the global rise in temperature associated with climate change. Heat stress in plants adversely affects not only the process of photosynthesis itself, but also leads to disturbances in plant growth and productivity, largely by exacerbating oxidative stress within plant cells [6].

The studies presented in this Special Issue [6] indicate that the application of low concentrations of salicylic acid can effectively enhance heat tolerance in cotton (*Gossypium hirsutum*) by, among other mechanisms, protecting photosynthetic pigments from heat-induced degradation.

## 6. Environmental Pollution as a Stress Factor Affecting Photosynthetic Efficiency

Environmental pollution is another factor that adversely affects the efficiency of photosynthesis, which may be particularly detrimental in the case of crop species such as *Zea mays* and *Lactuca sativa*. It has been demonstrated that contamination of irrigation water with cyanobacterial cultures and their associated toxins can lead to a reduction in net photosynthetic efficiency. Interestingly, researchers have shown that root exposure to water containing cyanobacterial toxins decreases total net photosynthesis, yet does not impair the electron transport processes within photosystem II.

Moreover, the presence of cyanobacteria in the irrigation medium also results in a decline in Rubisco activity and a reduction in total chlorophyll content in plant leaves [7].

## 7. Light Intensity, Nutrient Availability, and Conservation Physiology

Research on the relationship between photosynthetic efficiency, light intensity, and the availability of essential mineral nutrients such as nitrogen also provides valuable insights for developing strategies aimed at the conservation of endangered plant species. An illustrative example is the analysis of the interaction between light intensity and nitrogen supply in relation to photosynthetic performance and stress tolerance in *Picea neoveitchii* Mast. [8]. A key practical aspect of this study lies in its contribution to establishing a scientific basis for the protection and ecological restoration of endangered plant populations.

## 8. Light Spectral Quality and Its Impact on Plant Development

Light spectra quality is an important environmental factor determining plant growth and development. The roles of red and far-red light in the initiation of flowering are well established. However, considerably less is known about the role of blue light in this process. Nevertheless, blue light may also play an important role in the regulation of floral transition [9]. An insightful review by Kong and Zheng [9], published in this Special Issue, provides a comprehensive overview of the signaling network of blue light–mediated floral transition in plants, offering a critical analysis of the involved photoreceptors and key components of the blue-light-induced signal transduction pathway.

In turn, the practical use of combined blue and red-light spectra for supplemental lighting in spinach cultivation demonstrates that proper ratios of these wavelengths can promote favorable biomass accumulation. Moreover, the conducted studies have revealed the crucial role of phytochrome, the red-light photoreceptor, in regulating plant growth, as well as the importance of blue-light photoreceptors in mediating amino acid and sugar accumulation [1].

## 9. Conclusions

Light is a fundamental environmental factor influencing plant growth and developmental processes. The perception and utilization of light in plants occur at multiple levels and involve various receptors and regulatory mechanisms. Light governs not only vegetative growth but also the transition from the vegetative to the reproductive phase of development. These processes are mediated by specific red- and blue-light photoreceptors.

At the same time, blue and red light are absorbed by photosynthetic pigments within chloroplasts, driving the process of photosynthesis. This process, being directly dependent on the intensity of light reaching the leaves, is highly sensitive to fluctuations in light intensity. Consequently, over the course of evolution, plants have developed a range of mechanisms enabling them to acclimate to dynamically changing light environments.

All these aspects, along with their practical implications for crop cultivation, are addressed in the present Special Issue, which explores diverse topics within the broad field of plant photobiology.

## Data Availability

All papers cited in this editorial are available in the following link: https://www.mdpi.com/journal/plants/special_issues/L2986O6H6P (accessed on 24 October 2025).
